# Differential Refractive Index Sensor Based on Coupled Plasmon Waveguide Resonance in the C-Band

**DOI:** 10.3390/s21237984

**Published:** 2021-11-30

**Authors:** Qian Yang, Laixu Gao, Changwei Zou, Wei Xie, Canxin Tian, Zesong Wang, Feng Liang, Yihong Ke, Xinmei Zhou, Songquan Li

**Affiliations:** College of Physical Science & Technology, Lingnan Normal University, Zhanjiang 524048, China; yangq@lingnan.edu.cn (Q.Y.); gaolaixu@lingnan.edu.cn (L.G.); changweizou@hotmail.com (C.Z.); xiewei@lingnan.edu.cn (W.X.); cxtian@lingnan.edu.cn (C.T.); zswang@lingnan.edu.cn (Z.W.); liangf@lingnan.edu.cn (F.L.); kyh2932949211@163.com (Y.K.); ameria108@163.com (X.Z.)

**Keywords:** coupled plasmon waveguide resonance, differential intensity interrogation, refractive index sensor, C-band

## Abstract

We proposed a differential fiber-optic refractive index sensor based on coupled plasmon waveguide resonance (CPWR) in the C-band. The sensor head is a BK7 prism coated with ITO/Au/ITO/TiO_2_ film. CPWR is excited on the film by the S-polarized components of an incident light. The narrow absorption peak of CPWR makes it possible to realize dual-wavelength differential intensity (DI) interrogation by using only one incident point. To implement DI interrogation, we used a DWDM component to sample the lights with central wavelengths of 1529.55 and 1561.42 nm from the lights reflected back by the sensor head. The intensities of the dual-wavelength lights varied oppositely within the measurement range of refractive index, thus, a steep slope was produced as the refractive index of the sample increased. The experimental results show that the sensitivity is 32.15/RIUs within the measurement range from 1.3584 to 1.3689 and the resolution reaches 9.3 × 10^−6^ RIUs. Benefiting from the single incident point scheme, the proposed sensor would be easier to calibrate in bio-chemical sensing applications. Moreover, this sensing method is expected to be applied to retro-reflecting SPR sensors with tapered fiber tip to achieve better resolution than wavelength interrogation.

## 1. Introduction

A surface plasmon resonance (SPR) sensor is very sensitive to the change of refractive index of the measured medium. Due to the advantages of SPR sensors, such as high sensitivity, real-time monitoring, and label-free, until now, the research of SPR sensors and their application in bio-molecular interactions and chemical analysis is still a hot spot [1,2,3,4,5]. To make full use of the advantages of optical fiber sensing technology, various SPR sensors based on optical fiber have been developed. From the role of optical fiber in the sensor, fiber SPR sensors could be classified into sensing type [6,7] (using fiber as the coupling element) and light transmission type [8,9] (fiber is only used to transmit optical signal). Unlike the former, the latter usually used a non-fiber coupling element. Therefore, although the sensing structure is not compact, it is more robust because the sensor head is not as fragile as bare fiber tip. This type of sensor can employ either wavelength interrogation or intensity interrogation. When the working band is in the narrow C-band, the intensity interrogation would be a better choice because it does not need to consider the extremely wide SPR spectrum [10].

In previous work, a fiber-optic SPR remote sensor in the C-band has been demonstrated [11]. To deal with the influence of the power fluctuation of a light source on the resolution, the sensor is designed to work in the differential intensity (DI) interrogation mode. A BK7 prism coated with Au/TiO_2_ films serves as a sensor head, in which the thickness of TiO_2_ films is different. To enable two channels for DI interrogation, two fiber collimators were glued onto the sensor head. As a result, the sensor head should be large enough to ensure the space required to implement the scheme of two incident points. Therefore, the sensor head has little potential for miniaturization. Moreover, when the scheme of two incident points is applied to biochemical sensing, there would be another problem. Anisotropic or non-uniform surface refractive index perturbation is the most frequent situation in biochemical sensing applications of SPR sensors [12]. If the DI interrogation is used to measure one certain biosensing interaction on the surface of the multiple sensing area, there would be different deviation caused by the different distributed refractive index perturbation of the sensor, making the sensor difficult to calibrate. As a result, in the practical application of DI interrogation, a scheme of single incident point is preferred. Aiming to realize DI interrogation using a scheme with a single incident point in the C-band, two different wavelengths of light are needed, and the resonant spectrum should be narrow enough. Fano resonance based on various artificial nanostructures can provides narrow-band absorption or radiation spectrum. The high quality factor of Fano resonance is attractive in the field of ultrasensitive sensing and filtering [13,14,15]. It is also a good scheme to obtain a desired resonant spectrum with an appropriate width by using the traditional layered structure, since the preparation of the layered structure is not complex benefiting from the mature coating technique.

Four different SPR modes have been proposed as conventional SPR (CSPR), waveguide-coupled SPR (WCSPR), long-range SPR (LRSPR), and coupled plasmon waveguide resonance (CPWR). Unlike CSPR, which uses a single metal film, WCSPR is usually constructed by inserting a dielectric waveguide layer between two metal films. In angular interrogation, it exhibits two dips that are provided by SPR mode and waveguide mode; thus, it is convenient to accurately determine the thickness and dielectric constant of the functional layer in a biosensor [16]. Nevertheless, in wavelength interrogation, the spectrum of WCSPR would widen with the red shift of the working band just like the spectrum of CSPR. Therefore, the spectrum of WCSPR in the C-band would be too broad to be used by us. The structure that excites LRSPR is generally composed by substrate/dielectric layer/metal film/analyte [17]. When the refractive indexes of the dielectric layer and the analyte are equal or similar, the propagation distance of the surface plasmon is longer than that of CSPR, which means lower loss of LRSPR as well. As a result, the LRSPR exhibits a very sharp dip in angular interrogation [18], it also possesses a narrower spectrum under wavelength interrogation than that of the CSPR. The sensor base on LRSPR has the advantage of higher sensitivity compared with the sensor based on CSPR, a sensitivity of 5.9 × 10^4^ nm/RIUs to bulk refractive index change can be achieved [19]. It is possible to design a layer structure to obtain a sufficient narrow LRSPR spectrum in the C-band and ultra-high sensitivity can be expected. However, if the working band is limited within the narrow C-band (~35 nm), the measurement range would be severely compressed to an unacceptable level. What is more, the tuning of the measurement range becomes inconvenient since the performance of the LRSPR relies upon the existence of a symmetric environment.

CPWR mode is a mode supported by a waveguide usually composed of prism/noble metal film/dielectric/analyte [16], which fulfills the mode equation. The mode equations of P- and S-polarized component are different due to the different formulas of reflection coefficient [20]. The guided modes are related to waveguide parameters. Therefore, the CPWR mode can be excited by the P- or S- polarized component via adjusting the parameters of the film in the waveguide. Compared with the CSPR and WCSPR, the CPWR exhibits a narrower resonant spectrum, which could improve the detection resolution, but for us, the narrow spectrum makes it possible to realize dual-wavelength DI interrogation in the C-band using a single incident point. Commonly, CPWR devices’ sensitivity is 10-times less than that of the CSPR devices, but various approaches can be used to enhance the sensitivity [21], such as employing the coupling effect of the propagating SPR and localized SPR [22] or utilizing new sensitivity-enhancing materials [23]. It is worth mentioning that with the increase of the thickness of the overlayer, the CPWR resonant dip shifts to a longer wavelength and the corresponding sensitivity increases [24]. From the perspective of measurement range, if the working band is fixed within the narrow C-band, a sensor based on CPWR can obtain a larger measurement range than the one based on LRSPR because of its lower sensitivity.

In this work, we proposed a differential fiber-optic refractive index sensor based on CPWR in the C-band. The sensor head is a BK7 prism coated with ITO/Au/ITO/TiO_2_ film, enabling CPWR of S-polarized components of an incident light. An unpolarized C-band light emerging from a fiber collimator strikes upon the film twice and excites CPWR. The reflected light is coupled back to the collimator. A DWDM module samples the reflected light with central wavelengths of 1529.55 and 1561.42 nm for DI interrogation. The experimental results show that the intensities of the dual-wavelength lights vary oppositely within the measurement range from 1.3584 to 1.3689, which demonstrates that it is feasible to use a single incident point to implement DI interrogation in the C-band.

## 2. Preparation of Sensor Head

The plasmonic response of a sensing film is vital for the preparation of a sensor based on CPWR in the C-band. Therefore, we use a measurement apparatus we built up [11] to characterize the sensing film. In the apparatus, the original photodetectors (ThorLabs, Newton, NJ, USA, DET50B) were replaced by the detectors (ThorLabs DET20C2) with much lower dark current to facilitate measurement. BK7 glass substrates (Agar Scientific Ltd., Essex, UK) were used to prepare the sensing layer. An ITO/Au/ITO film was prepared by sputtering a pure gold target and an ITO target (In_2_O_3_:SnO_2_ = 9:1) using two ion-sputtering equipment (SBC-12, KYKY Technology Co., Ltd., Beijing, China). One is used for Au film deposition and the other is used for ITO film deposition. The target-substrate distance of each ion sputtering equipment is set to 50 mm. Under the condition of argon pressure of 5 Pa and current of 3 mA, the gold film was sputtered for 100 s. For both ITO films, the pressure of argon is 5 Pa, the current is 5 mA, and the sputtering lasted for 60 s. TiO_2_ film were capped on ITO/Au/ITO film by using e-beam evaporation at a rate of 0.3 nm/s. The thickness of TiO_2_ film was monitored by a quartz crystal film-thickness monitor during evaporation. We chose ITO film as an adhesion layer because ITO has much smaller extinction coefficient than Cr [25,26]. The stability of the sensing film was tested by immersing the coated substrates into water for one week. It is found that ITO/Au/ITO/TiO_2_ film is stable, while Au/TiO_2_ film fell off from the substrate and the TiO_2_ film of ITO/Au/TiO_2_ film showed wrinkles after one day.

**Figure 1:**
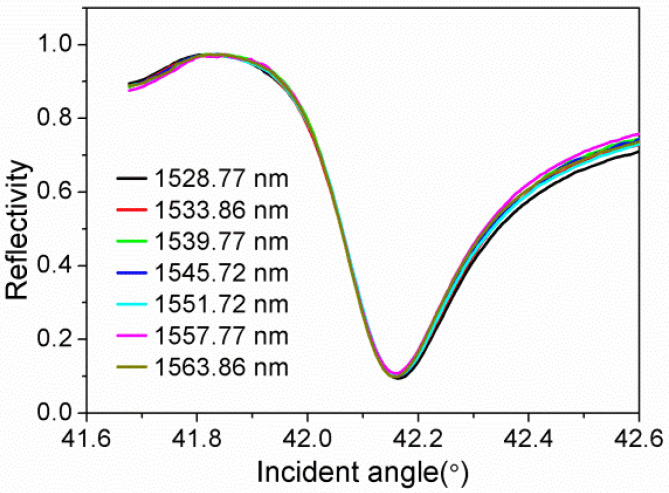
SPR curves of ITO/Au/ITO film in the C-band.

The SPR curves *R*(*θ*) of ITO/Au/ITO film were collected at several incident wavelengths across the C-band using the established apparatus. As shown in Figure 1, these curves are very close to each other, and there is only a slight difference in resonant angles as well as corresponding reflectivities, which is attributed to the dispersion characteristics of Au film in the C-band [27]. By fitting the SPR curves, the calculated effective thickness ranges from 33 to 35 nm, in which the thickness corresponding to the incident light wavelengths of 1528.77 and 1563.86 nm is 33 nm. Therefore, we considered that it is reasonable to estimate the thickness as 33 nm, and then we fitted the SPR curves again to determine the effective dielectric constants when the thickness is fixed at 33 nm.

**Figure 2:**
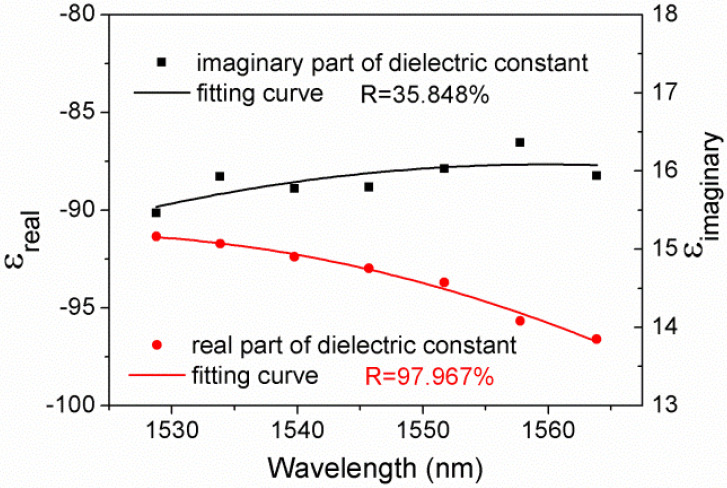
Dielectric constants of ITO/Au/ITO film in the C-band.

As shown in Figure 2, there is a lager deviation between the value of imaginary part of dielectric constant and the fitting curve, compared with the case of real part ones. This is due to the measurement accuracy of the apparatus, specifically, the repeated positioning accuracy of the rotary stage is 0.005°, which is comparable to the interval of angular scanning (~0.006°). As a result, the measurement accuracy of resonant angle and reflectivity is limited. Even so, the fitting results still show a certain degree of regularity.

**Figure 3:**
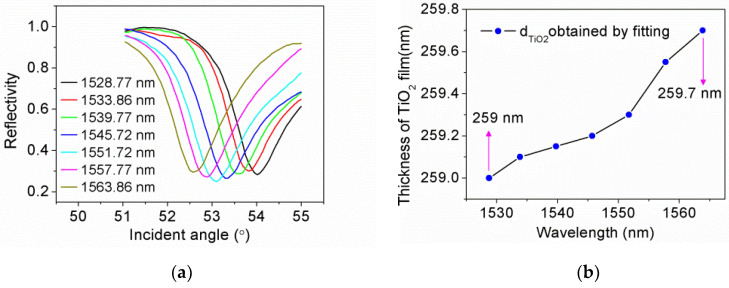
(**a**) The CPWR curves of ITO/Au/ITO/TiO_2_ film in the C-band; (**b**) The calculated thickness of TiO_2_ film.

The incident light was adjusted to S-polarization by rotating the Glan–Taylor polarizer. The CPWR curves *R*(*θ*) of ITO/Au/ITO/TiO_2_ film were obtained in air. As depicted in Figure 3a, owing to the dispersion of TiO_2_ film, the resonance angle decreases gradually with the increase of wavelength. Utilizing the parameters of ITO/Au/ITO film determined previously and assuming the dielectric constants of TiO_2_ film conform to the values in the reference [28], we fitted the CPWR curves and calculated the thickness of TiO_2_ film corresponding to each incident light wavelength. As can be seen in Figure 3b, the calculated thickness of TiO_2_ film is distributed between 259 and 259.7 nm. For simplicity, we determined the thickness of TiO_2_ film as 259.35 nm.

**Figure 4:**
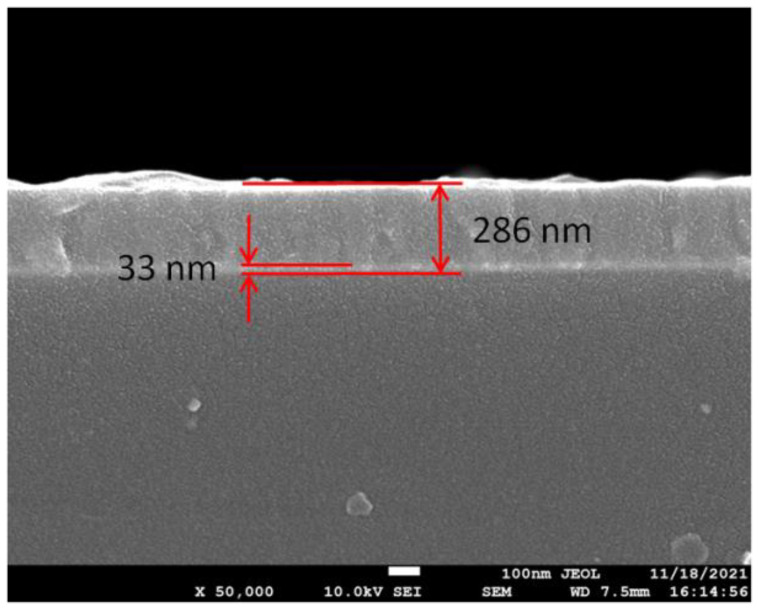
The cross section of the ITO/Au/ITO/TiO_2_ film.

We characterized the cross section of the film by using a SEM (FEI Sirion IMP). The image is shown in Figure 4. The thickness of ITO/Au/ITO film agrees well with the calculated one. The thickness of TiO_2_ film is 253 nm, which is about 6 nm less than the calculated value. Nevertheless, the thickness error of 2% can also show that the calculation results have a certain reliability. We performed simulation to verify the ITO/Au/ITO/TiO_2_ film is able to support CPWR. The incident angle is fixed at 68.26°, and the parameters of the sensing film determined previously are adopted. Multilayer Transfer Matrix Method for both P- and S-polarized light is utilized to simulate twice interactions between light and sensing film when the refractive index of sample (n_s_) is 1.359. For comparison, the SPR response of 60 nm Au/28.7 nm TiO_2_ film (denoted as film1) and CPWR response of 60 nm Au/539 nm TiO_2_ film (denoted as film2) excited by P-polarized component were also simulated. In the simulation, the dispersion of Au and TiO_2_ film are sourced from reference [27,28], respectively.

**Figure 5:**
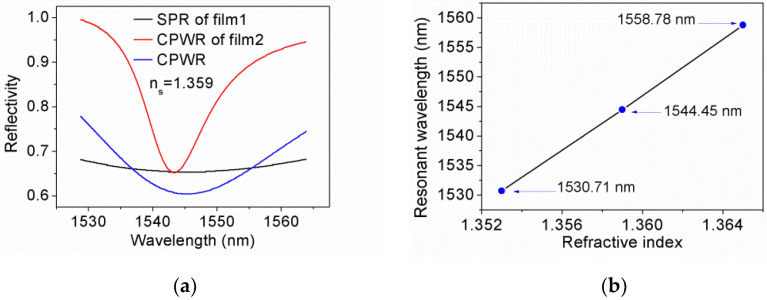
(**a**) The simulation of CPWR for ITO/Au/ITO/TiO_2_ film (blue line), SPR for 60 nm Au/28.7 nm TiO_2_ film (black line), and CPWR for 60 nm Au/539 nm TiO_2_ film (red line); (**b**) The relationship between the resonant wavelength and n_s_ for ITO/Au/ITO/TiO_2_ film.

The results are shown in Figure 5a, suggesting that: (1) conventional SPR spectrum of 60 nm Au/28.7 nm TiO_2_ film is too broad to be used for DI interrogation; (2) the CPWR spectrum of 60 nm Au/539 nm TiO_2_ film is narrower than the others. To ensure the linearity of DI response, the light of two wavelengths corresponding to full width at half maximum of a spectrum should be selected for DI operation. Due to the narrow spectrum, higher sensitivity can be expected, but the measurement range would be reduced. To expand the range, the light of several wavelengths are needed additionally for DI operation, This segmented detection method requires more detectors, which will also increase the cost. (3) CPWR of ITO/Au/ITO/TiO_2_ film can be excited by the S-polarized component of an incident light. Even though the spectrum is broader than the one of film2, it is sufficient to realize DI interrogation by using a scheme of single incident point and dual-wavelength lights. Figure 5b illustrates the resonant wavelength versus n_s_ for ITO/Au/ITO/TiO_2_ film, from which a sensitivity of wavelength interrogation can be derived as 2338 nm/RIUs within a measurement range of 1.353–1.365.

**Figure 6:**
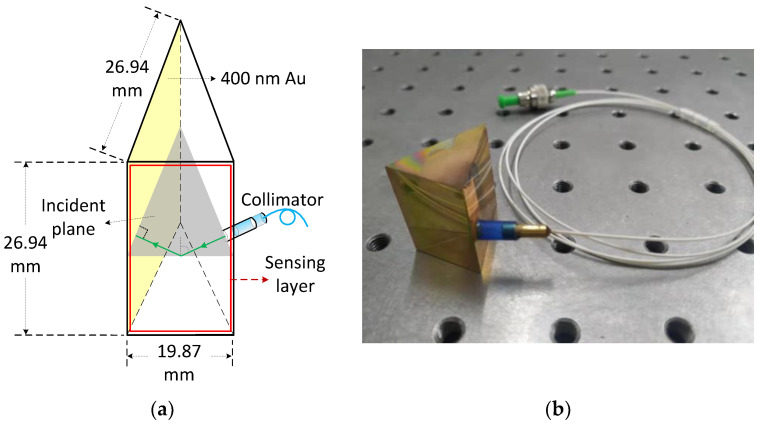
(**a**) Structural diagram of the sensor head; (**b**) Photograph of the sensor head.

A BK7 prism was customized which should have an isosceles-triangular base of angles of 68.26°, corresponding to the cross-section dimensions 27 mm × 27 mm × 20 mm and height 27 mm. It should be mentioned that to ensure more accurate incident angle, we customized a larger prism instead of compact one, which is enough to prove the feasibility of the proposed sensor. Nevertheless, we measured the size of the prism with a Vernier caliper, and found that the cross-section size of the prism is 26.94 mm × 26.94 mm × 19.87 mm; thus, the actual value of the incident angle would be 68.36°. We deposited a thick Au film (~400 nm in thickness) onto a square facet of prism as a reflective coating, and then prepared ITO/Au/ITO/TiO_2_ film on the rectangular facet of prism. The fixing process of an fiber collimator and prism has been described in our previous work [11], but unlike before, we only use one fiber collimator. A rigid pipe is used to connect the prism and collimator. All parts in contact with each other were fixed with UV curing adhesive. The structural diagram and photograph of the sensor head are shown in the Figure 6.

## 3. DI Sensing Based on CPWR and Discussions

**Figure 7:**
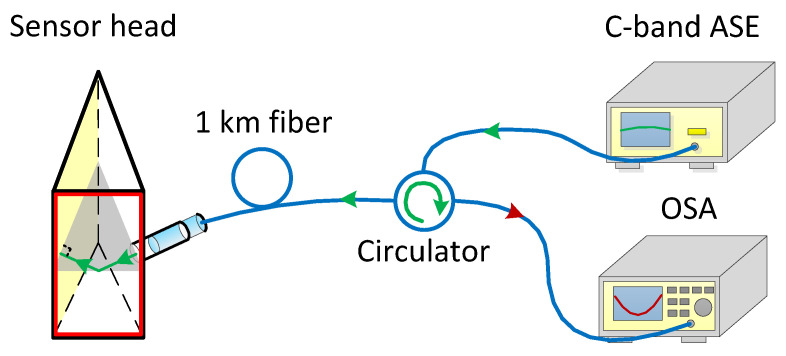
Structure diagram of the testing system for CPWR response of the sensor head.

We established a testing system to investigate the CPWR response of the prepared sensor head in frequency domain. The testing system is shown in Figure 7. A light beam from a C-band ASE source (ASE-C-100-T-B, Hefei Max-ray Photoelectric Technology Co., Ltd., Hefei, China) enters the input port of a circulator and exits from the collimator after passing through a fiber (G.652) with a length of one kilometer. The light emerging from the collimator enters the prism at normal incidence, and then excites CPWR on the sensing layer at an incident angle of 68.36°. After the light reflected off the sensing layer is reflected by the 400-nm gold film, its incidents upon the sensing layer again at the same incident angle and excites the CPWR one more time. The light output from the prism couples back to the collimator. The reverse transmitted light passes through the circulator and is received by an optical spectrum analyzer (OSA, Anritsu MS9710C, Anritsu, Atsugi, Japan) with a resolution of 50 pm. For testing, the sensor head was immersed into the prepared aqueous solutions of glycerol. To avoid the influence of temperature fluctuation on the measurement as possible, the temperature of solution was controlled at 25 ± 0.2 °C.

**Figure 8:**
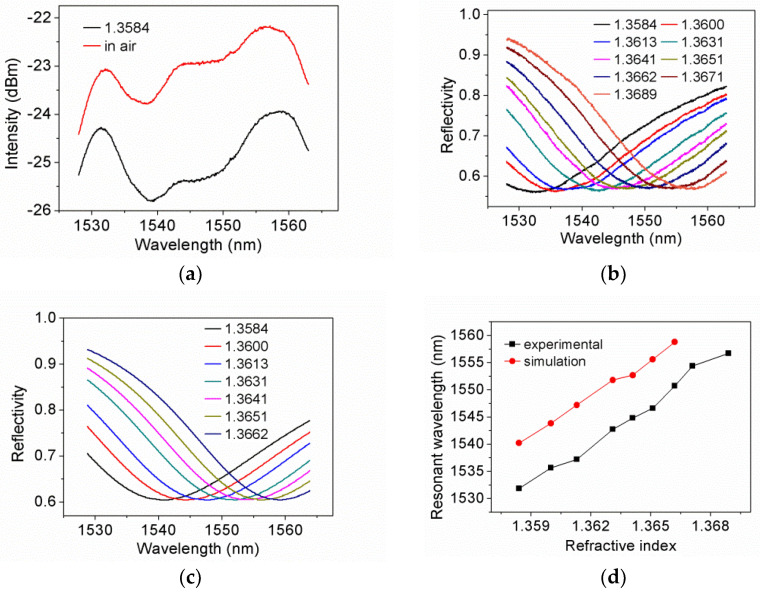
(**a**) CPWR response at n_s_ = 1.3584; (**b**) Normalized CPWR spectrums at different n_s_; (**c**) Simulated CPWR spectrums at different n_s_; (**d**) Experimental and simulated resonant wavelength at n_s_.

Figure 8a shows the CPWR spectrum of the sensor head at n_s_ = 1.3584. The reference spectrum for normalization was measured when the sensor head was in air. It is obvious from the figure that absorption has occurred, but hardly determine the position of the resonant wavelength, which is due to the spectrum of ASE is not flat enough. Figure 8b shows the normalized CPWR spectrums at different n_s_. From Figure 8b, we can get the following information: Firstly, the resonant wavelength increases from 1531.85 to 1556.70 nm as n_s_ increases from 1.3584 to 1.3689, suggesting that the sensitivity is 2366 nm/RIUs within a measurement range of 1.3584~1.3689. The sensitivity is 1.6-times that of 1396.85 nm/RIUs reported in the literature [21], which is the benefit of moving the working wavelength into the C-band. Compared with the sensitivity of 8000 nm/RIUs reported in the literature [23], the sensitivity of the proposed sensor is more than three-times lower, owing to the absence of sensitivity enhancement mechanism. Secondly, we can use the light with central wavelengths of about 1530 and 1560 nm for DI interrogation.

For comparison, we simulated the CPWR spectrums within the measurement range, and extracted resonant wavelength at n_s_ from both simulated and experimental spectrums, the results are shown in Figure 8c,d. It is illustrated that the resonant wavelength of the simulated spectrum is about 8-nm larger than that of the experimental spectrum in the measurement range. The discrepancy mainly comes from the thickness of TiO_2_ film on the sensor head is a few nanometers smaller than that of TiO_2_ film used to determine the film parameters, which is due to the TiO_2_ films not being deposited in the same batch.

**Figure 9:**
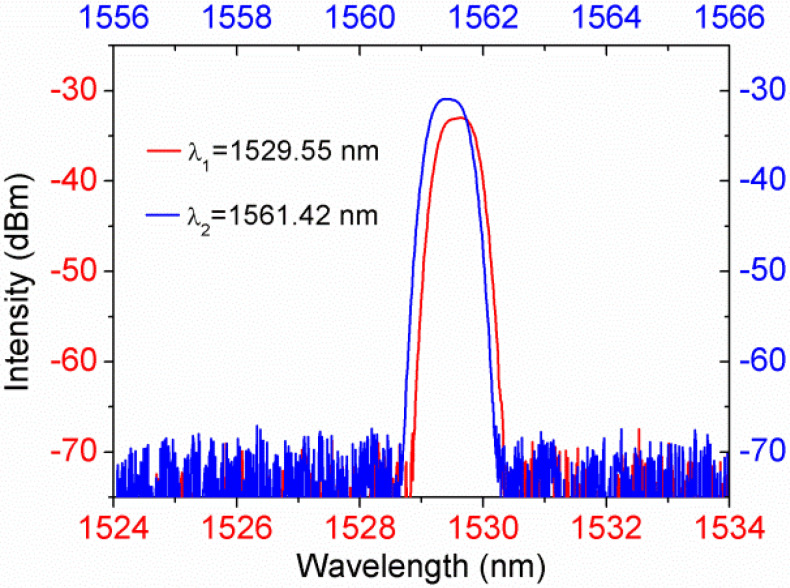
Spectrums of DWDM’s two channels.

**Figure 10:**
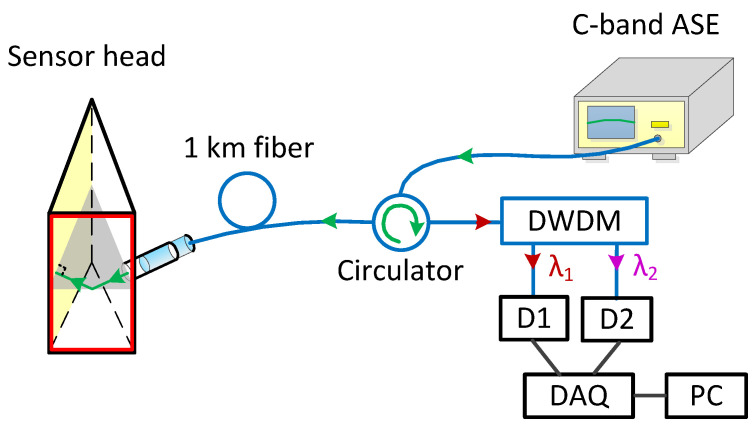
Structure diagram of DI sensing system.

To implement DI interrogation, we used a DWDM component (48CH 100 GHz AAWG module, Hefei Max-ray Photonics Co., Ltd., Hefei, China) to sample the reflected light with central wavelengths of 1529.55 and 1561.42 nm. The two wavelengths are named λ_1_ and λ_2_, respectively. Figure 9 shows the spectrums of the DWDM’s two channels. The intensity of λ_2_ is higher than that of λ_1_ due to the characteristics of the ASE spectrum as depicted in Figure 8a. We replaced the optical spectrum analyzer with a DWDM component, and the DI sensing system is shown in Figure 10. The DWDM samples the lights with central wavelengths of λ_1_ and λ_2_ from the output light of the circulator. The dual-wavelength lights were received by two photodetectors (ThorLabs, DET01CFC). A synchronous DAQ module with an actual accuracy of 15-bits collect the voltage signals for DI interrogation. At each n_s_, 50 data were recorded by collecting one data every 2 s. The normalized differential signal is obtained by dividing the difference in voltage by the sum of voltages, expressed as:

(1) $$Signal_{normalized}\;=\;\left(V_{\lambda 1}-V_{\lambda 2}\right)/\left(V_{\lambda 1}+V_{\lambda 2}\right)$$

**Figure 11:**
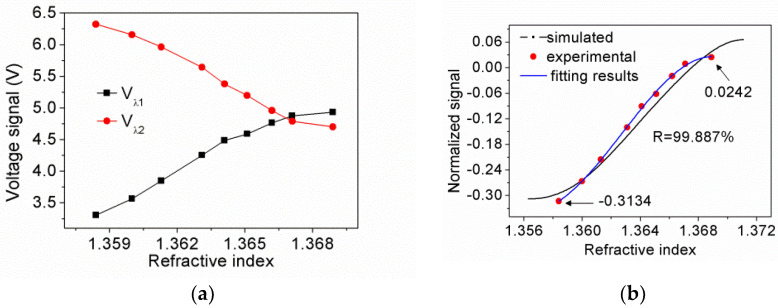
(**a**) Voltage signals of dual-wavelength lights at n_s_; (**b**) Normalized signals including simulated signal, experimental data, and fitting results.

Figure 11a shows the $V_{\lambda 1}$ and $V_{\lambda 2}$ at each n_s_. As expected, within the measurement range, $V_{\lambda 1}$ dropped down as $V_{\lambda 2}$ rose up because the reflectivity of light at the corresponding wavelength also experiences such a change trend. Figure 11b depicts the experimental and simulated DI signal. The experimental results agree well with the simulated ones, however, there is some discrepancy. Because the dielectric constants of the layer affect the DI signal through the effect on the width and depth of the CPWR spectrum, we consider that the discrepancy mainly comes from the deviation between the dielectric constants of ITO/Au/ITO film and the real value. Due to the simulated signal is not linear natively, we used third-order polynomial fitting to analyze the experimental data, The relation between the experimental signal and the n_s_ changing exhibits good regularity, the fit goodness coefficient is R = 99.887%. The experimental signal rose from −0.3134 to 0.0242 as the n_s_ increases from 1.3584 to 1.3689; thus, the average sensitivity of the sensor can be derived as 32.15/RIUs which is close to that of 31.7/RIUs in our previous work [11].

**Figure 12:**
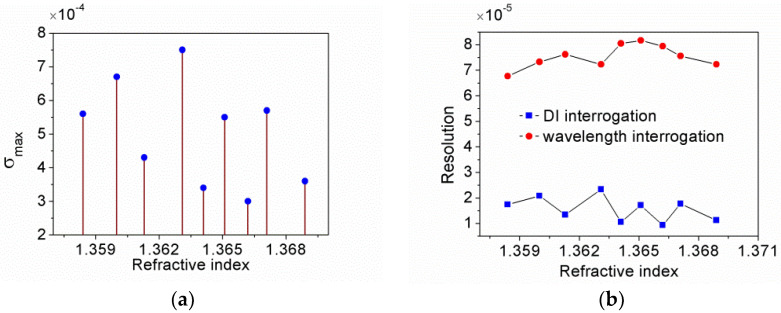
(**a**) Maximum fluctuation of the normalized signal at n_s_; (**b**) Resolution of DI interrogation and wavelength interrogation.

The ultimate resolution of the proposed sensor is directly limited by the maximum fluctuation of the normalized signal which can be expressed as $\sigma_{\max}$. Figure 12a illustrated $\sigma_{\max}$ at each n_s_. $\sigma_{\max}$ is distributed between 3.0 × 10^−4^ and 7.5 × 10^−4^. According to the sensitivity of 32.15/RIUs, the resolution are derived at each n_s_, as shown in Figure 12b. The lowest resolution is 2.33 × 10^−5^ RIUs reaches the typical value of intensity interrogation, the highest one is 9.3 × 10^−6^ RIUs.

For comparison, we evaluated the resolution of the sensor head for wavelength interrogation by using the formula in the literature [29]. It is found that the resolution of DI interrogation and wavelength interrogation are in the same order of magnitude. However, several factors affect the resolution of wavelength interrogation, such as the sensitivity, signal to noise ratio (SNR) associated with amplitude noise, full width at half maximum (FWHM) of CPWR spectrum and optical resolution of 50 pm provided by the optical spectrum analyzer. As a consequence, the resolution limit of resonant wavelength is about 0.18 nm according to our evaluation, which is about three-times larger than 50 pm, causing the deterioration of the sensor’s resolution. For wavelength interrogation, the best resolution is 6.77 × 10^−5^ RIUs. There may be two approaches to improve the resolution by reducing the amplitude noise of spectrum, one is that a light source with very stable power should be used, another is that the CPWR and reference spectrum should be recorded simultaneously. The former would lead to a high cost of the sensing system, while the latter is not easy to realize by using an optical spectrum analyzer with only one channel. It is worth noting that the DI sensor base on CPWR exhibits better resolution than the sensor of wavelength interrogation, even using devices with much lower cost than an optical spectrum analyzer.

## 4. Conclusions

In this work, we presented a differential fiber-optic refractive index sensor based on CPWR in the C-band. The sensor head is a BK7 prism coated with ITO/Au/ITO/TiO_2_ film, which can excite CPWR utilizing S-polarized components of an incident light. The bandwidth of CPWR is suitable for DI interrogation using only a single incident point and dual-wavelength lights. To implement DI interrogation, we used a DWDM module to sample the lights with central wavelengths of 1529.55 and 1561.42 nm from the lights reflected back by the sensor head. The experimental results showed that the reflectivity of the dual-wavelength lights vary oppositely within the measurement range; thus, a steep slope was produced as n_s_ varies. The sensitivity is 32.15/RIUs in the n_s_ range of 1.3584 to 1.3689 and the resolution reaches 9.3 × 10^−6^ RIUs. The single incident point scheme of the sensor head is expected to provide great convenience for calibration in bio-chemical sensing applications. The structure of the sensor head is quite simple; thus, a compact sensor head could be manufactured for practical applications. What is more, the approach of DI interrogation using CPWR in the C-band has potential to be applied in retro-reflecting SPR sensors with tapered fiber tip to achieve better resolution than wavelength interrogation.
